# T-cell regulation in Erythema Nodosum Leprosum

**DOI:** 10.1371/journal.pntd.0006001

**Published:** 2017-10-09

**Authors:** Edessa Negera, Stephen L. Walker, Kidist Bobosha, Rawleigh Howe, Abraham Aseffa, Hazel M. Dockrell, Diana N. Lockwood

**Affiliations:** 1 London School of Hygiene and Tropical Medicine (LSHTM), Faculty of Infectious Tropical Diseases, London, United Kingdom; 2 Armauer Hansen Research Institute (AHRI), Addis Ababa, Ethiopia; University of Tennessee, UNITED STATES

## Abstract

Leprosy is a disease caused by Mycobacterium leprae where the clinical spectrum correlates with the patient immune response. Erythema Nodosum Leprosum (ENL) is an immune-mediated inflammatory complication, which causes significant morbidity in affected leprosy patients. The underlying cause of ENL is not conclusively known. However, immune-complexes and cell-mediated immunity have been suggested in the pathogenesis of ENL. The aim of this study was to investigate the regulatory T-cells in patients with ENL. Forty-six untreated patients with ENL and 31 non-reactional lepromatous leprosy (LL) patient controls visiting ALERT Hospital, Ethiopia were enrolled to the study. Blood samples were obtained before, during and after prednisolone treatment of ENL cases. Peripheral blood mononuclear cells (PBMCs) were isolated and used for immunophenotyping of regulatory T-cells by flow cytometry. Five markers: CD3, CD4 or CD8, CD25, CD27 and FoxP3 were used to define CD4^+^ and CD8^+^ regulatory T-cells. Clinical and histopathological data were obtained as supplementary information. All patients had been followed for 28 weeks. Patients with ENL reactions had a lower percentage of CD4^+^ regulatory T-cells (1.7%) than LL patient controls (3.8%) at diagnosis of ENL before treatment. After treatment, the percentage of CD4^+^regulatory T-cells was not significantly different between the two groups. The percentage of CD8^+^ regulatory T-cells was not significantly different in ENL and LL controls before and after treatment. Furthermore, patients with ENL had higher percentage of CD4^+^ T-ells and CD4^+^/CD8^+^ T-cells ratio than LL patient controls before treatment. The expression of CD25 on CD4^+^ and CD8^+^ T-cells was not significantly different in ENL and LL controls suggesting that CD25 expression is not associated with ENL reactions while FoxP3 expression on CD4^+^ T-cells was significantly lower in patients with ENL than in LL controls. We also found that prednisolone treatment of patients with ENL reactions suppresses CD4^+^ T-cell but not CD8^+^ T-cell frequencies. Hence, ENL is associated with lower levels of T regulatory cells and higher CD4^+^/CD8^+^ T-cell ratio. We suggest that this loss of regulation is one of the causes of ENL.

## Author summary

Leprosy reactions (Type 1 and 2) are important causes of nerve damage and illness. Erythema Nodosum Leprosum (ENL) also called type 2 reaction is a severe systemic immune-mediated complication of borderline and lepromatous leprosy. ENL causes high morbidity and thus requires immediate medical attention. We recruited 77 untreated patients with lepromatous leprosy (46 patients with ENL reactions and 31 patients without ENL reactions) in Ethiopia to better define the immune regulation process in patients with ENL reactions. We took blood samples at 3 time points (before, during and after prednisolone treatment) and measured regulatory T-cells at each time point. Patients with ENL reactions had a lower percentage of CD4^+^ regulatory T-cells than in non-reactional LL patient controls before treatment. Patients with ENL reactions had higher percentage of CD4^+^ T- cells and CD4^+^/CD8^+^ ratio than LL patient controls before treatment. These experiments indicate the need to explore ways of restoring regulatory T-cells in patients with ENL reactions to control the undesired outcome of the reaction.

## Introduction

Leprosy is a disease caused by *Mycobacterium leprae*, an intracellular acid-fast bacillus. It mainly infects the skin and peripheral nerves. Leprosy is a spectrum of disease with a five-district forms with the localized tuberculoid leprosy (TT) and the generalized lepromatous leprosy (LL) forming the two poles of the spectrum. The clinical spectrum of leprosy correlates with the host immune response [1].

Leprosy reactions (Reversal reactions and Erythema Nodosum Leprosum) are immune-mediated inflammatory complications of the disease which can occur before, during or after successful completion of multi-drug treatment (MDT) [2]. They are a major cause of morbidity in a significant proportion of leprosy patients [3]. Erythema Nodosum Leprosum (ENL) is an inflammatory complication of leprosy, manifesting as tender erythematous skin lesions and systemic features of disease including fever, neuritis and bone pain [4].

ENL reactions were initially thought to be due to immune-complex deposition in the blood vessels suggestive of Arthus reaction [5]. However, immune-complex deposition is not consistently demonstrable and typical features of immune-complex diseases are absent in ENL.

Histologically, neutrophils are considered the signature cell in ENL lesions [6]. However, not all clinically confirmed ENL cases have neutrophilic infiltration in lesions [7]. Hence, there is little direct evidence as to the actual role of neutrophils in the pathogenesis of ENL.

Recent data suggest that cell-mediated immune responses may also play an important role in the pathogenesis of ENL [8, 9]. An increased percentage of CD4^+^ T-cells and reduced CD8^+^ T-cells in ENL lesions and in the periphery were reported previously [10, 11]. Increased mitogen induced lymphoproliferation and inhibition of strong antigen-induced leukocyte migration during ENL reaction had been reported in ENL patients [12]. However, others reported that reduced percentage of CD4^+^ and increased percentage of CD8^+^ T-cells in these patients [8] Hence, strong evidence is required to explain the role of T- cells in the pathophysiology of ENL.

Regulatory T-cells (T_regs_) formerly called suppressor T-cells are subpopulations which modulate the immune system and maintain tolerance to self-antigens [13]. It has previously been described that T_regs_ inhibit naïve CD4^+^ T-cell proliferation and differentiation, prevent cytotoxic activity of CD8^+^ T-cells, suppress the activation and antibody production of B-cells, and limit the stimulatory capacity of antigen presenting cells by down regulating the surface expression of costimulatory molecules such as CD80 and CD86 [14].

A reduced percentage of T_regs_ has been associated with immune-complex mediated autoimmune diseases such as Wegener’s granulomatosis (WG) [15] and anti-neutrophil cytoplasmic antibody (ANCA)-associated vasculitis [16]. In patients with these diseases, the percentage of T_regs_ is inversely related to disease progression or relapse and a relatively increased proportion of T_regs_ is associated with rapid disease remission. However, the role of T_regs_ in leprosy has only been addressed by few studies [17–20] and a clear picture has not yet emerged.

Although there are few studies on the association of T_reg_ phenotypes with leprosy, *M. leprae* specific suppression of effector responses had been described prior to the definition and characterisation of T_regs_ [21]. Mehra et al. made the first report when they described suppression of proliferative responses to concanavalin A in the presence of lepromin in LL and BL patients [22].

Quantification of Tregs in PBMCs stimulated with *M. leprae* antigenic preparations and phytohemagglutinin (PHA) by flow cytometry and in the skin lesions by immunohistochemistry showed that M. leprae antigens induced low lymphoproliferative responses (low mean cell counts per minute) but higher number of T_regs_ in lepromatous patients than in tuberculoid patients (TT) [23]. A cell subset analysis and confocal microscopy of skin biopsies in Ethiopian leprosy patients showed increased frequencies of T_regs_ in the blood as well as in the lesions of LL patients compared to TT and borderline leprosy lesions [18]. Similar results have been reported in Indian [24, 25].

The analysis of the frequency of circulating T_regs_ in PBMCs of 6 ENL patients by flow cytometry showed that both the absolute count and percentage of T_regs_ were significantly lower in patients with ENL reaction (1.2%) compared to patients with LL 2.8% [17, 26]. However, the authors also reported that patients with ENL had significantly higher percentage of T_regs_-FoxP3 expression along with higher percentages of effector T-cells than patients with the other types of leprosy.

A recent study performed flow cytometry on PBMCs isolated from 6 patients with ENL in comparison to 8 LL patient controls, after stimulation with *M. leprae* sonicated antigen (MLSA) described a significant reduction in percentage of CD4^+^CD25^+^FoxP3^+^T_regs_ and Mean Fluorescence Intensity of FoxP3 in PBMC of ENL patients [20]. However, the same study also reported an increased expression of FoxP3 in the PBMCs of patients with ENL compared to LL controls when measured by qPCR.

Th17 cells have been identified as a new subset of the T- helper cells and as potential mediators of inflammation associated with various autoimmune and mycobacterial diseases [27]. Th17 cells are the least studied T-cells in leprosy and only three studies have indicated the involvement of Th-17 in immunopathogenesis of ENL [20, 26, 28]. Th-17 produces a group of cytokines called IL-17 of which IL-17A is one of the groups. IL-17A is an immunoregulatory cytokine capable of promoting the generation of pro-inflammatory cytokines and chemokines, which leads to the attraction of neutrophils and macrophages to the inflammation site (Jin and Dong, 2013). Recently a cross-sectional study has reported that increased IL-17A production to *M.leprae* stimulation in ENL patients compared to non-reactional LL patients [20].

The conflicting reports could be due to use of small sample size, lack of strict case definitions, use of inappropriate controls and variations in assay methods. Nonetheless, these studies of immune regulation draw our attention to the potential importance of regulatory T-cells in the evolution and subsequent course of ENL reactions, though a consensus conclusion remains elusive. Therefore, in the current study we investigated the frequency of T_regs_ in a relatively large cohort of patients with ENL reactions compared to non-reactional LL matched controls before, during and after prednisolone treatment of ENL cases.

## Materials and methods

### Ethics statement

Informed written consent for blood and skin biopsies were obtained from patients following approval of the study by the Institutional Ethical Committee of London School of Hygiene and Tropical Medicine, UK, (#6391), AHRI/ALERT Ethics Review Committee, Ethiopia (P032/12)and the National Research Ethics Review Committee, Ethiopia (#310/450/06). All patient data analysed and reported anonymously.

### Study design

A case-control study with follow-up for 28 weeks after the initiation of prednisolone treatment was used to recruit 46 patients with ENL reaction and 31 non-reactional LL patient controls between December 2013 and October 2015 at ALERT Hospital, Ethiopia.

### Clinical case definitions

The clinical assessment of the patient was used as main diagnostic criterion for ENL cases and LL controls [4]

#### Erythema Nodosum Leprosum (ENL)

ENL was clinically diagnosed when a patient with lepromatous or borderline lepromatous leprosy had tender subcutaneous erythematous skin lesions and systemic features of disease such as fever, neuritis and bone pain. Other accompanying systemic features included joint pain, bone tenderness, orchitis, iritis, oedema malaise, anorexia and lymphadenopathy.

#### Lepromatous leprosy (LL)

Lepromatous leprosy was clinically diagnosed when a patient had widely disseminated nodular lesions with ill-defined borders and Bacterial index (BI) above two.

#### Acute ENL

Acute ENL was defined as an ENL episode clearing within 24 weeks of prednisolone treatment.

#### Chronic ENL

Chronic ENL was defined as an ENL occurring for 24 weeks or more either during which a patient has required ENL treatment continuously or where any treatment free period has been 27 days or less.

#### Recurrent ENL

Recurrent ENL was defined as a second or subsequent episode of ENL occurring 28 days or more after stopping or steady decrease of prednisolone treatment for ENL.

#### ENL recurrence or flare-up

ENL recurrence or flare-up **w**as defined as the appearance of new ENL nodules after initial control, either whilst on treatment or after 28 days off treatment.

#### New ENL case

New ENL case was defined as the occurrence of ENL for the first time in a patient with lepromatous leprosy.

### Demographic and clinical data collection

A structured questionnaire was used to obtain clinical data for each participant. The ENL International Study (ENLIST) format was modified and used for clinical data recording. The data collection sheet included the demographic, clinical and diagnostic information set following the standard guideline at each time point. The clinical information included core points such as the clinical feature, skin lesion, nerve functions and systemic involvement.

### Clinical sample collection

Blood samples were obtained from each patient at three time points: at recruitment before prednisolone administration, after 12 and 24 weeks of prednisolone treatment for ENL cases. The 12th week was chosen as second sampling time point because the steady decrease in prednisolone dose reaches less than half of the start dose by week 12 and after the 24^th^week prednisolone is normally off unless the patient experiences a recurrence or chronic condition. The third time-point (24th week) sample was obtained when an ENL patient completed prednisolone treatment and the treatment free period has lasted 15 days or more. This means the third sample was obtained 15 days after the patient stopped prednisolone treatment to avoid the effect of steroids on immunological assays.

### PBMC isolation, freezing and thawing

Twenty milliliter of venous blood was collected in sterile BD heparinised vacutainer tubes (BD, Franklin, Lakes, NJ, USA). PBMC were separated by density gradient centrifugation at 800g for 25 min on Ficoll-Hypaque (Histopaque, Sigma Aldrich, UK) as described earlier[18]. Cells were washed three times in sterile 1x phosphate buffered saline (PBS, Sigma Aldrich, UK) and re-suspended with 1mL of Roswell Park Memorial Institute (RPMI medium 1640 (1x) + GlutaMAX + Pen-Strip GBICO, Life technologies, UK). Cell viability was determined by 0.4% sterile Trypan Blue solution (Sigma Aldrich, UK) ranged from 94–98%. PBMC freezing was performed using a cold freshly prepared freezing mediumcomposed of 20% Foetal Bovine Serum (FBS, heat inactivated, endotoxin tested ≤5 EU/ml, GIBCO Life technologies, UK), 20% dimethyl sulphoxide (DMSO) in RPMI medium 1640 (1x). Cells were kept at -80°C for 2–3 days and transferred to liquid nitrogen until use. Cell thawing was done as described [29]. The procedure is briefly described as: cells were transported in liquid nitrogen to a water bath (37°C) for 30 to 40 seconds until thawed half way and resuspended in 10% FBS in RPMI medium 1640 (1x)(37°C)containing 1/10,000 benzonase until completely thawed, washed2 times (5–7 minutes each) and counted. The percentage viability obtained was above 90%. Cell concentration was adjusted to 10^6^ cells/mL in RPMI, 1 ml of the cell suspension was added to wells of a 24 well polystyrene cell culture plate (Corning Costar cell culture plates), and the plates incubated at 37°C in a 5% carbon dioxide incubator. After overnight rest [30], cells were stained for flow cytometry with fluorochromes conjugated antibodies as described below.

### Surface and intracellular staining for flow cytometry

The cells were harvested, transferred to round bottomed FACS tubes (Falcon, BD, UK) and washed twice at 400g for 5 minutes at room temperature. The cells were resuspended in 50μl of PBS and incubated in 1ml of 10% human AB serum (Sigma Aldrich, UK) for 10 minutes in the dark at room temperature to block nonspecific Fc-mediated interactions, and centrifuged at 400g for 5 minutes. After resuspending cells in 50μL PBS buffer, Life/dead staining was performed at a concentration of 1μl /1mL live/dead stain (V500 Aqua, Invitrogen, Life technologies, UK) for 15 minutes at 4°C in the dark. Cells were washed once and stained for surface markers directed against CD3-Pacific blue, CD4-APC-eFluor 780, CD8-PerCp-Cy5.5, CD127-APC, CD161-PE (all eBioscience, UK) and CD25-PE-Cy7 (BD, Biosciences, UK). After staining at 30 minutes at 4°C, the cells were washed with FACS buffer. One mL of 1x FoxP3 Fixation/Permeabilization buffer (eBioscience, UK) was added to each tube, mixed thoroughly and incubated at 4°C for 60 minutes and washed with permeabilization buffer at 400g for 5 minutes. Cells were resuspended in 50μl of buffer followed by staining the permeablized cells with anti-human FoxP3 (FITC, eBioscience) for 30 minutes at 4°C. After two additional washes, the cells were re-suspended in 400μl FACS buffer for acquisition. Compensation beads were stained in parallel with samples under the same environment. A single-stained OneComb eBeads (affymetrix, eBioscience, UK) for all fluorescence compensation except for the live dead stain were used. For the viability dye, cells rather than beads were stained and used for fluorescence compensation.

### Sample acquisition and gating strategy

Forward scatter height (FSC-H) versus Forward scatter area (FSC-A) plots were used to select singlets, and FSC-A versus dead cell marker plots identified viable cells. Side scatter area (SSC-A) versus FSC-A plots were used to discriminate lymphocytes from monocytes and residual granulocytes. The threshold for FSC was set to 5,000. For each sample, 500,000–1,000,000 cells were acquired. S1 Fig)

Flow cytometry analysis was performed with FlowJo version 10 (Tree Star, USA) using logicle (bi-exponential) transformations as recommended [31, 32]. CD4^+^ and CD8^+^ T_regs_ were defined as CD3^+^CD4^+^CD25^+^FoxP3^+^CD127^lo/-^ (S1 Fig) and CD3^+^CD8^+^CD25^+^FoxP3^+^CD127^lo/-^ (S2 Fig) cells, respectively [33]. CD25+ and FoxP3+ cells were also gated on CD4 and CD8 T-cells (S3 Fig). The percentage of each subpopulation was defined relative to the parent population and data exported to Excel for each sample, compiled and exported to statistical software for further analysis.

### Statistical analysis

Differences in percentage of T-cell subsets were analyzed with either the two-tailed Mann-Whitney U test or the Wilcoxon signed rank non-parametric tests using STATA 14 version 2 (San Diego California USA). Graphs were produced by GraphPad Prism version 5.01 for Windows (GraphPad Software, San Diego California USA). The median and Hodges–Lehmann estimator were used for result presentation. Hodges–Lehmann is used to measure the effect size for non-parametric data [34]. P-values were corrected for multiple comparisons. The statistical significance level was set at p≤0.05.

## Results

We described the median percentage of T_regs_ and other T-cell subtypes before, during and after completion of prednisolone treatment of patients with ENL reactions and compared to the corresponding non-reactional LL patient controls as well as within ENL patients. Comparison between cases and controls were used to investigate the association of T-cell subsets with ENL reactions and comparison within ENL was used to describe the kinetics of these T-cell subsets in response to prednisolone treatment.

### Patient clinical background

Forty-six LL patients with ENL reaction and 31 LL patient controls without ENL reaction were recruited between December 2013 and October 2015. The male to female ratio was 2:1 with a median age of 27.5 [range: 18–56] years in patients with ENL and 3:1 with a median age of 25.0 [range: 18–60] years in patients with non-reactional LL controls. All ENL patients were untreated with corticosteroid before recruitment. At time of recruitment, 20 ENL patients were previously untreated with MDT, 21 were on MDT and 5 were completed MDT treatment. Twenty non-reactional LL patients were about to start MDT, 7 were on MDT and 4 were completed MDT at recruitment.

### ENL is associated with loss of T-cell regulation

By using CD4^+^CD25^+^FoxP3^+^ CD127^-/lo^ and CD8^+^CD25^+^FoxP3^+^ CD127^-/lo^ as CD4^+^ and CD8^+^Treg markers respectively, we investigated the median percentage of CD4^+^ and CD8^+^Tregs in patients with ENL reaction compared to matched non-reactional LL patient controls before, during and after prednisolone treatment of ENL patients. We also described the kinetics of these cells within ENL group before, during and after treatment.

**Fig 1:**
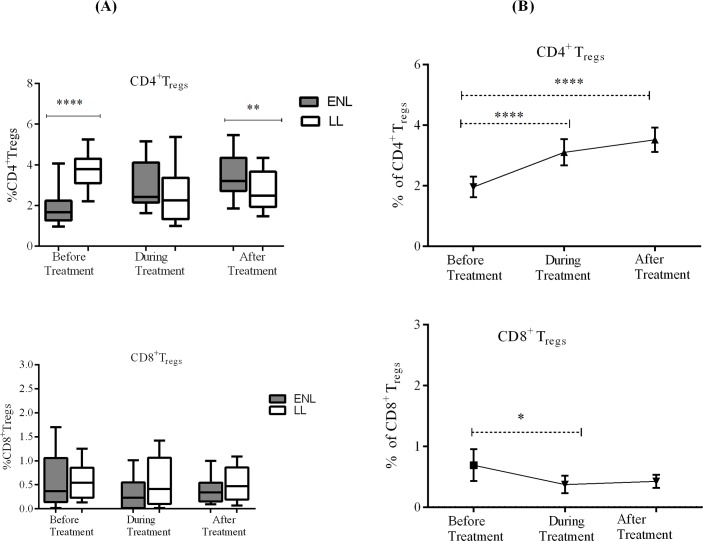
**Median percentage of CD4^+^ and CD8^+^ regulatory T- cells** (CD3^+^CD4^+^CD25^+^FoxP3^+^CD127^-/lo^ and CD3^+^ CD8^+^CD25^+^FoxP3^+^CD127^-/lo^ respectively): (A) in patients with ENL and LL controls before, during and after treatment, (B) within ENL patients before, during and after prednisolone treatment. * P≤0.05; ** P≤ 0.0.005; *** P< 0.001; **** P< 0.0001. Box and whiskers (A) and Error bars (B) show median ± interquartile range.

The median percentage of CD4^+^ regulatory T- cells was significantly lower (1.67%) in the PBMCs of patients with ENL compared to LL patient controls (3.79%) before treatment (P≤0.0001; ΔHL = 1.93%). During treatment, the percentage of CD4^+^ T_regs_ in the PBMCs of patients with ENL almost doubled (from 1.67% to 3.21%) while it significantly dropped from 3.79% to 2.43% in LL patient controls, though these differences did not reach statistical significance. After treatment, 3.2% of CD4^+^ T-cells were positive for these T_regs_ markers in patients with ENL while only 2.5% of CD4^+^ T-cells were positive in LL patient control (P≤0.005). The median percentage of CD8^+^T_regs_ was lower in the PBMCs of untreated patients with ENL (0.37%) compared to LL patient controls (0.54%) but the difference was not statistically significant (P ≥ 0.05). During treatment, the median percentage of CD8^+^T_regs_ decreased in both groups (0.23% in patients with ENL and 0.42% in LL patient controls) (P ≥ 0.05). The median percentage of CD8^+^T_regs_ after treatment in patients with ENL and LL controls was 0.34% and 0.47% respectively (P ≥ 0.05). Thus, it appears that CD4^+^T_regs_ are associated with ENL reaction but not CD8^+^T_regs_ (Fig 1A).

Comparison within ENL group has shown that the median percentage of CD4^+^ regulatory T- cells was significantly lower (1.67%) in the PBMCs of patients with ENL reactions before treatment than during treatment (2.5%) (P<0.0001; ΔHL = 1.0%). After treatment, the median percentage of CD4^+^T_regs_ increased to 3.20% and it was significantly higher than before treatment (1. 67%) (P<0.0001; ΔHL = 1.6%) suggesting that ENL reaction is associated with decreased percentage of CD4^+^T_reg_ cells. In contrast to CD4^+^ T_regs_, the percentage of CD8^+^ T_regs_ was not significantly different before and after prednisolone treatment of patients with ENL reactions (Fig 1B).

### Increased CD4^+^ T- lymphocytes in untreated ENL patients

**Fig 2:**
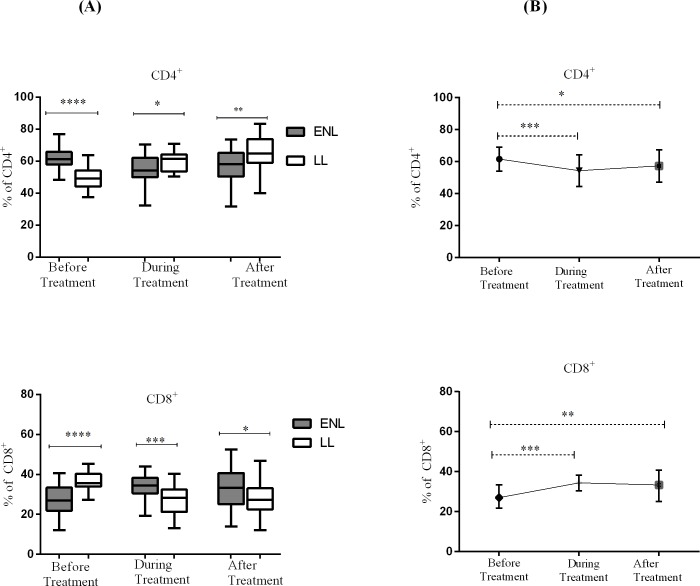
Median percentage of CD4^+^ and CD8^+^ T-cells: (A) in patients with ENL and LL controls before, during and after treatment, (B) within ENL patients before, during and after treatment. * P≤0.05; ** P≤ 0.0.005; *** P< 0.001; **** P< 0.0001. Box and whiskers (A) and Error bars (B) show median ± interquartile range.

Untreated patients with ENL reactions had a significantly higher median percentage of CD4^+^T-cells (61.3%) compared to LL patient controls (49.1%) at enrolment (P < 0.0001; ΔHL = 12.8%). During prednisolone treatment of patients with ENL reactions, the median percentage of CD4^+^ T cells decreased to 54.2% while that of LL patient controls increased to 61.4% and the difference was statistically significant (P≤0.05). On the other hand, patients with ENL had a significantly lower median percentage of CD8^+^ T-cells (27.0%) before treatment compared to LL patient controls (35.7%) and the difference was statistically significant (P<0.0001; ΔHL = 8.2%). Interestingly, while patients with ENL were on treatment, unlike the CD4^+^ T-cells, the median percentage of CD8^+^ T-cells increased to 34.4% and it was higher than that of LL patient controls (28.3%) (P<0.001). After treatment, the corresponding values did not significantly changed in patients with ENL (33.5%) and LL controls (27.2%) (Fig 2A).

Comparison within ENL group revealed that the median percentage of CD4^+^ T-cells was significantly higher (61.3%) before treatment than during treatment (54.2%) (P≤0.001; ΔHL = 6.8%) and after treatment (P≤ 0.05; ΔHL = 4.6%) (P>0.05). On the other hand, the median percentage CD8^+^ T-cells in untreated ENL patients was significantly lower (27.0%) than during treatment (34.4%) (P≤0.0001; ΔHL = 6.8%) and after treatment (P≤0.005) (Fig 2B).

### Untreated ENL patients had higher CD4^+^/CD8^+^ T-cell ratio

**Fig 3:**
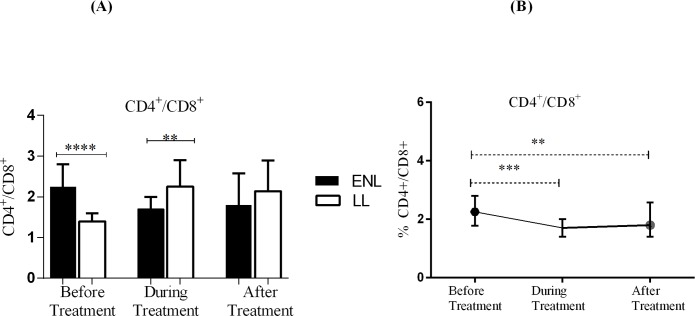
Median percentage CD4^+^ / CD8^+^ T-cells ratio: (A) in patients with ENL and LL controls before, during and after treatment, (B) within ENL patients before, during and after treatment. ** P≤ 0.0.005; *** P< 0.001; **** P< 0.0001. Box and whiskers (A) and Error bars (B) show median ± interquartile range.

The CD4^+^/CD8^+^ T-cell ratio was higher (2.3: 1) in patients with ENL compared to LL patient controls (1.4:1) before treatment (P<0.001; ΔHL = 0.09). However, a significantly lower CD4^+^/CD8^+^ T-cell ratio was seen in patients with ENL (1.7: 1) compared to LL patient controls (2.25:1) during treatment (P≤ 0.001). After treatment, the ratio of CD4^+^/CD8^+^ T- cell was 2.14: 1 and 1.8:1 in patients with ENL and LL controls respectively and the difference was not statistically significant (P>0.05) (Fig 3A). Hence, this result indicates that patients with ENL reactions and non-reactional LL controls not only showed significant differences in the percentage of CD4^+^ and CD8^+^ T-cells but also in their CD4^+^ / CD8^+^ T-cell ratio. Patients with ENL had higher median percentages ratio of CD4^+^ and CD4^+^/CD8^+^ T-cells than LL patient controls before treatment.

Analysis within ENL group has shown that the median percentage of CD4^+^ to CD8^+^ T-cell ratio before treatment was significantly higher (2.23:1) than during treatment (1.8: 1) (P<0.0001; ΔHL = 0.6) and after prednisolone treatment (1.7:1) (P≤0.005; ΔH = 0.5) (Fig 3B). This finding shows that prednisolone treatment was associated with a decreased median percentage of CD4^+^ T-cells and CD4^+^ to CD8^+^ T-cell ratio but with increased median percentage of CD8^+^ T-cells in ENL reactions.

### Increased IL-17 producing T-cells are associated with ENL reaction

**Fig 4:**
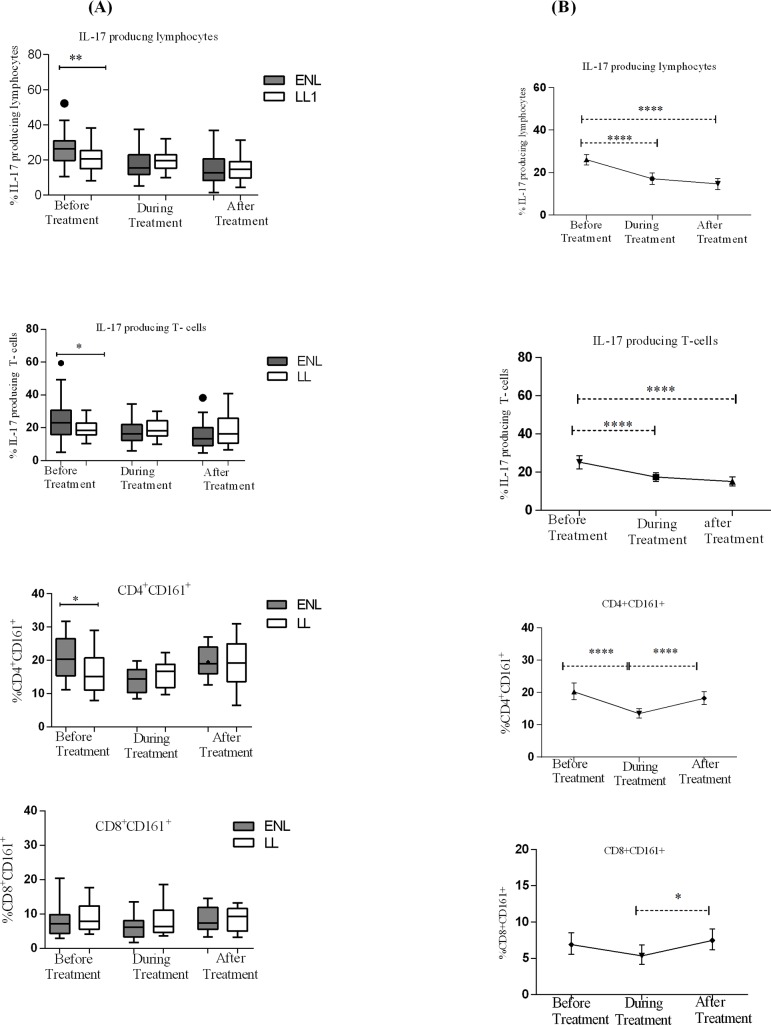
Median percentage of IL-17 producing cells: (A) in patients with ENL and LL controls before, during and after treatment, (B) within ENL patients before, during and after treatment. * P≤0.05; ** P≤ 0.0.005; *** P< 0.001; **** P< 0.0001. Box and whiskers (A) and Error bars (B) show median ± interquartile range.

Patients with ENL had a significantly higher median percentage of IL-17 producing lymphocytes (26.45%) than LL patient controls (20.6%) before treatment (P≤ 0.05; ΔHL = 5.7). Similarly, the proportion of IL-17 producing T-cells in the PBMCs of patients with ENL was considerably higher (23.1%) than in LL patient controls (18.4%) before treatment and the difference was statistically significant (P≤ 0.05; ΔHL = 4.5). Patients with ENL had a higher percentage of IL-17 producing CD4^+^ T-cells than LL patient controls before treatment. The percentage of IL-17 producing CD8^+^ T-cells was not significantly different in both patient groups (Fig 4A).

The median percentage of IL-17 producing cells was also compared within patients with ENL reactions before, during and after prednisolone treatment to see the trend of these cells during the follow-up period. The median percentage of IL-17 producing lymphocytes in the PBMCs of untreated patients with ENL reactions was significantly higher (26.45%) than during treatment (15.4%) (P<0.0001, ΔHL = 9.5%). After treatment, the median percentage of these IL-17 producing lymphocytes was further decreased to 12.7% and it was significantly lower than before treatment (P<0.0001, ΔHL = 11.855%). Similarly, the proportion of IL-17 producing T-cells was substantially higher (23.1%) before treatment than during treatment (16.4%) and after treatment (13.2%) (P<0.0001) (Fig 4B). It was found that the proportion of IL-17 producing CD4^+^ T-cells was significantly decreased during treatment (P< 0.0001) but again increased after treatment. Unlike IL-17 producing lymphocytes, the median percentage of IL-17 producing CD4^+^ T-cells was not significantly different before and after treatment showing that CD4^+^ T-cells may not be the main source of IL-17 or IL-17 producing CD4+ -cells may not respond to prednisolone treatment in these patients. IL-17 producing CD8^+^ T-cells did not show any significant difference before, during and after treatment of patients with ENL reactions (Fig 4B). Prednisolone treatment did not seem to affect the proportion of Il-17 producing CD8^+^ T-cells in ENL but it could affect transiently IL-17 producing CD4^+^T-cells.

### ENL patients and non-reactional LL patient controls had similar level of CD25 expression

**Fig 5:**
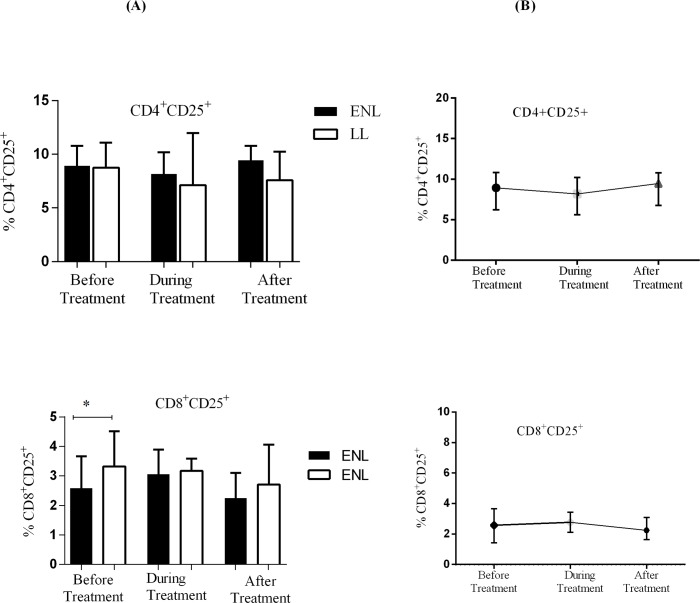
The median percentage of CD25 expression on CD4^+^ and CD8^+^ T-cells: (A) in patients with ENL and LL controls before, during and after treatment, (B) within ENL patients before, during and after treatment. * P≤0.05. Box and whiskers (A) shows median ± interquartile range.

The median percentage of CD25 positive T-cells in CD4^+^ T-cells in the PBMCs of untreated patients with ENL and LL patient controls were found to be 8.9% and 8.8% respectively (P>0.05). About 2.6% of CD8^+^ T-cells had expressed CD25 in the PBMCs from patients with ENL and a slightly higher proportion of CD8^+^ T-cells (3.2%) expressed CD25 in the PBMCs from LL patient controls before treatment (P≤0.05).The expression of CD25 on both CD4^+^ and CD8^+^ T- cells during and after treatment did not change in both patient groups (Fig 5A). Hence, these results indicate that the expression of CD25 by CD4^+^ and CD8^+^T-cells is similar in patients with ENL and LL controls and it does not discriminate active ENL from non-reactional LL.

The analysis of differential expression of CD25 by CD4^+^ T-cells within ENL group has revealed that the median percentage expression of CD25 in CD4^+^ T-cells before and during treatment was 8.9% and 8.2% respectively. After treatment with prednisolone, about 9.5% of CD4^+^ T-cells expressed CD25. The expression of CD25 by CD4^+^ T-cells before, during and after treatment was not statistically significantly different (P> 0.05) implying that prednisolone may not affect the expression of CD25 in CD4^+^ T-cells. Similarly, the expression of CD25 in CD8^+^ T-cells during and after prednisolone treatment of patients with ENL did not change (P> 0.05). About 2.6% of CD8^+^ T-cells were positive for CD25 staining before treatment. The median percentage of CD8^+^CD25^+^ T- cells during and after treatment was 3.2% and 2.2% respectively (P> 0.05) (Fig 5B).

### Decreased FoxP3 expression by CD4^+^ T-lymphocytes in untreated ENL patients

**Fig 6:**
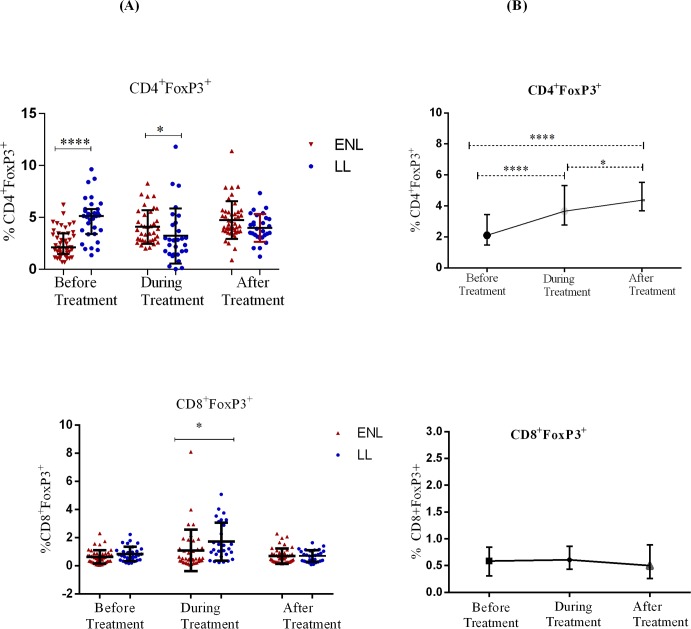
The median percentage of FoxP3 expression on CD4^+^ and CD8^+^ T-cells: (A) in patients with ENL and LL controls before, during and after treatment, (B) within ENL patients before, during and after treatment. * P≤0.05; **** P< 0.0001. Error bars show median ± interquartile range.

The median percentage of FoxP3-expressing CD4^+^ T-cells in the PBMCs of patients with ENL was lower (2.1%) compared to the median percentage expressed in the PBMCs of LL patient controls (5.1%) before treatment (P<0.0001). During treatment, the frequency of FoxP3-expressing CD4^+^ T-cells slightly increased to 3.5% in patients with ENL and decreased by half from 5.1% to 2.6% in LL controls and the difference between the two groups was statistically significant (p≤0.05). However, the frequency of FoxP3-expressing CD4^+^ T-cells in the PBMCs from patients with ENL and LL controls did not change significantly after treatment implying the possible association of reduced percentage of CD4^+^ FoxP3^+^ T-cells in patients with active ENL reaction. Although the median percentage of FoxP3-expressing CD8^+^ T-cells in the PBMCs of patients with ENL was slightly lower (0.57%) compared to LL controls (0.71%) before treatment, the difference was not statistically significant (P>0.05). During treatment, patients with ENL had a lower frequency of CD8^+^FoxP3^+^ T-cells (0.49%) compared to LL patient controls (1.17%) (P≤0.05). After treatment, the percentage of FoxP3 expression in CD8^+^ T-cells in patients with ENL and LL controls was not statistically significantly different suggesting that CD8^+^FoxP3^+^ T-cells may not associated with ENL reaction (P ≥0.05) (Fig 6A).

Comparison within ENL group has shown that the median percentage of CD4^+^FoxP3^+^ T-cells was significantly lower (2.1%) before treatment than during (3.5%) (P<0.0001; ΔHL = 1.4%) and after prednisolone treatment (4.4%) (P≤0.0001; ΔHL = 2.2). The expression of FoxP3 in CD4^+^ T-cells doubled after prednisolone treatment of patients with ENL suggesting an association of prednisolone and FoxP3 expression in CD4^+^ T-cells unlike the CD25 expression in these patients. Interestingly, the expression of FoxP3 in CD8^+^ T-cells was not significantly different before (0.59%), during (0.49%) and after (0.50%) prednisolone treatment of these patients (Fig 6B). Hence, it appears that prednisolone treatment does not affect the expression of FoxP3 in CD8^+^ T-cells unlike in CD4^+^T-cells.

### Lower frequency of CD25^+^ FoxP3^+^ double positive T-lymphocytes in active ENL patients than in LL patient controls

**Fig 7:**
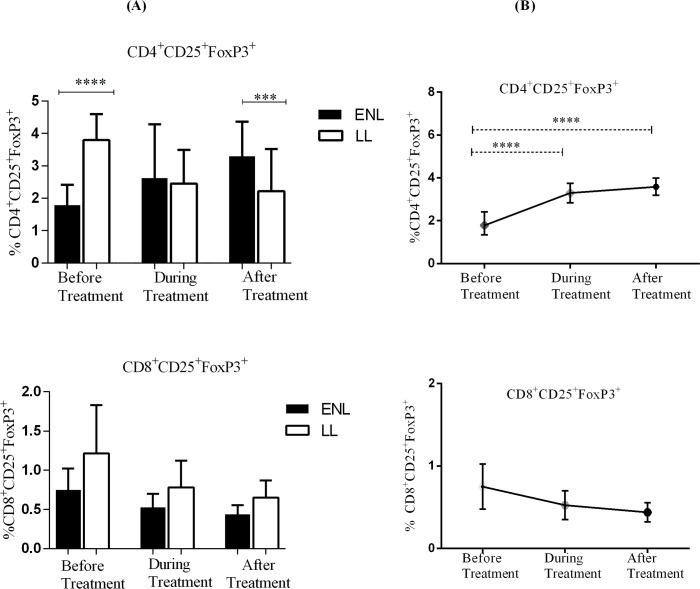
Median percentage of CD4^+^ and CD8^+^ T-cells expressing CD25^+^ FoxP3^+^ double positives: (A) in patients with ENL and LL controls before, during and after treatment, (B) within ENL patients before, during and after treatment. * P≤0.05; *** P< 0.001; **** P< 0.0001. Box and whiskers (A) and Error bars (B) show median ± interquartile range.

The frequency of CD25^+^FoxP3^+^ expression in CD4^+^ and CD8^+^ T-lymphocytes was measured in the PBMCs of patients with ENL and LL controls before, during and after treatment. About 1.8% of CD4^+^ T-cells expressed CD25^+^FoxP3^+^ in the PBMCs of untreated patients with ENL which was significantly lower than the proportion of CD25^+^FoxP3^+^ cells expressed in CD4^+^ T-cells (3.8%) in the PBMCs of LL patient controls (P<0.0001). During treatment, the median percentage of CD4^+^CD25^+^FoxP3^+^ T-cells in the PBMCs of patients with ENL increased to 2.6% while it decreased from 3.8% to 2.5% in LL patient controls. After treatment, the percentage of CD4^+^CD25^+^FoxP3^+^ T-cells in the PBMCs of patients with ENL had further increased to 3.3% while it dropped to 2.2% in patients with LL controls and the difference was statistically significant (P≤0.001) (Fig 7A). A small proportion of CD8^+^ T- cells in the PBMCs of patients with ENL and LL controls expressed CD25^+^FoxP3^+^. The percentage of CD8^+^CD25^+^FoxP3^+^ T- cells in the PBMCs of patients with ENL and LL controls was 0.4% and 0.6% respectively before treatment and slightly decreased in both groups (0.28% in patients with ENL and 0.52% in LL controls) during treatment. After treatment, these figures were slightly increased in both groups. The frequency of CD8^+^CD25^+^FoxP3^+^ T- cell expression in the PBMCs of patients with ENL and LL controls was not significantly different before, during and after treatment (Fig 7A). Hence, it appears that CD4^+^CD25^+^FoxP3^+^ T- cells are more closely associated with ENL reaction than do the CD8^+^CD25^+^FoxP3^+^ T- cells.

Analysis within ENL group has shown that the median percentage of CD4^+^CD25^+^FoxP3^+^ T-cells in the PBMCs of patients with ENL was significantly lower before treatment than during treatment (2.6%; P<0.0001; ΔHL = 1.04%) and after treatment (3.3%; P<0.0001; ΔHL = 1.55%). On the other hand, the median percentage of CD8^+^CD25^+^FoxP3^+^ T-cells was not significantly different before, during and after treatment (Fig 7B).

## Discussions

To describe the relative frequency of T_regs_ in the PBMCs from patients with ENL and LL controls, the markers CD3^+^CD4^+^CD25^+^FoxP3^+^CD25^+^CD127^-/lo^ were used for the definition of CD4^+^T_regs_ and CD3^+^CD8^+^CD25^+^FoxP3^+^CD25^+^CD127^-/lo^ for CD8^+^ T_regs_. Comparisons between ENL cases and LL patient controls were made to investigate the association of regulatory T-cells and other T-cell subsets with ENL reactions while comparisons within ENL group was made to provide information on the kinetics of T-cell regulation and other T-cell subsets in response to prednisolone treatment.

**Loss of T-cell regulation and increased CD4/CD8 T-cell ratio in active ENL:** in this study, the median percentage of CD4^+^T_regs_ in patients with ENL was significantly lower (1.7%) than in LL patient controls (3.8%) before treatment. In addition, after treatment, the percentage of CD4^+^T_regs_ in patients with ENL was increased to 3.2% while in LL patient controls it was decreased to 2.5%. T_regs_ are the least studied T-cells in patients with ENL. There are only two cross-sectional studies by the same authors, which had reported the reduced frequency of T_regs_ in patients with ENL compared to other clinical forms of the spectrum. The study by Attia et al. has reported 1.2% of T_regs_ in the whole blood of patients with ENL compared to 2.8% in LL controls although the study was included only 6 patients with ENL and 8 LL controls [17]. Another study by the same group in 2014 reported 1.2% and 2.8% of T_regs_ in the whole blood of 6 patients with ENL and 11 non-reactional LL controls respectively. Although these two studies are in agreement with the findings here, they reported slightly lower percentages of T_regs_ in both groups than we found. Unlike previous studies, we included large number of patients with ENL and non-reactional LL patient controls and they were followed for 28 weeks, and samples were obtained at three time points. We have also clearly set the case definitions for ENL before recruitment and used more markers to define T_regs_ which can be considered as one of the strengths of our study. Previous studies used CD4^+^CD25^high^ FoxP3^+^ as T_reg_ markers. However, a consensus on the thresholds of CD25 expression needed to delineate T_reg_ cells within the CD25^high^ population is difficult to attain, and variations in FoxP3 expression within the CD25^high^ population have been observed even in healthy individuals [35]. Hence, the definition of CD25 high and low is highly subjective which could lead to biased reports. In addition, many authors have also used healthy individuals and other clinical forms of leprosy such as TT and T1R as a basis for comparison with ENL, which leads to discordant results. Only patients with either BL or LL will develop ENL reactions. However, not all patients with BL or LL develop ENL. Hence, for immunological studies non-reactional either BL or LL patients are the appropriate controls for ENL patients.

On the other hand, in this study, the median percentage of CD8^+^T_regs_ was not significantly different in patients with ENL and LL controls before or after treatment. Hence, it appears that the development of ENL in patients with lepromatous leprosy is mainly associated with the decreasing frequency of CD4^+^T_regs_ being unable to balance the excessive immune activation via CD4^+^ T-cells. Studies have shown that T_regs_ inhibit naïve CD4^+^ T-cell proliferation and differentiation and hence, downregulate immune activation [14]. Similar findings have also been reported elsewhere in immune-complex mediated autoimmune diseases such as Wegener’s granulomatosis (WG) [15]) and Anti-neutrophil cytoplasmic antibody (ANCA)-associated vasculitis [36, 37].

Comparison within ENL group has shown that untreated patients with ENL cases had significantly lower percentage of CD4^+^T_regs_, which was considerably increased after prednisolone treatment. On the other hand, the frequency of CD8^+^ T_regs_ did not show any significant difference before and after prednisolone treatment. Thus, our finding shows that CD4^+^T_regs_ are implicated in ENL reactions but not CD8^+^Tregs. Comparison of our finding with previous studies was not possible since our study is the first case-control follow up study comparing the kinetics of T_regs_ at different time points in individual patients with ENL. However, similar findings have been reported in several independent studies elsewhere in autoimmune diseases. Nevertheless, it is not reasonable to make comparisons with autoimmune diseases since the aetiology of the ENL is different from that of autoimmune diseases. Studies in infection models described the significant immunosuppressive activity of T_regs_ for antigen-specific T-cells. T_regs_ are considered critical for suppressing immune responses to self-antigens and preventing autoimmunity and for regulating immunity to foreign antigens, especially those derived from pathogens that establish persistent infections [38].

### Increased CD4^+^ T- lymphocytes in untreated ENL patients

The results indicated that patients with ENL had a significantly higher median percentage of CD4^+^ T-cells and lower CD8^+^ T-cells before treatment compared to LL controls. Patients with ENL had also a higher CD4/CD8 ratio (2.3:1) compared to LL controls (1.4:1). Prednisolone treatment significantly reduced the percentage of CD4^+^ T-cells in patients with ENL implying that prednisolone could suppress CD4^+^ T-cells to resolve the inflammation. Other studies elsewhere have shown that prednisolone treatment promotes an immunological state that favours immune regulation rather than inflammation through downregulation of CD4^+^ T-cell proliferation [39]. The CD4^+^/CD8^+^ ratio obtained in our study for patients with ENL was higher than the reference value for apparently healthy Ethiopian adults (1.5:1) [40]. However, the CD4^+^/CD8^+^ ratio of LL patient controls was slightly lower than the normal value. Hence, it is logical to conclude that rather than the actual percentage of CD4^+^ and CD8^+^ T cells, the balance between the two T-cell subtypes is indeed associated with ENL reactions. Therefore, the higher the CD4^+^/CD8^+^ ratio the greater is the risk of developing ENL reactions. Increased CD4^+^ counts and CD4^+^/CD8^+^ T-cells ratio in 11 Indian ENL patients has been reported [41] which is in agreement with the present finding. However, they did not look at the proportion of CD4^+^ T-cells and CD4^+^/CD8^+^ ratio during and after treatment unlike the present study. Similar results have been reported by several studies [10–12, 42, 43]. Lymphocyte imbalance (CD4^+^and CD8^+^) has also been indicated in the progression of many other diseases elsewhere [44, 45].

However, in addition to the CD4^+^/CD8^+^ T-cell ratio, other factors such as the expression pattern of costimulatory molecules on T-cells (CD28) and on antigen presenting cells (CD80/86) in patients with ENL need to be explored. Abnormal T-cell co-simulation and T-cell senescence have been implicated in the expansion of effector memory T-cells and are thought to facilitate the breakdown of tolerance in inflammatory diseases such as anti-neutrophil cytoplasmic autoantibodies (ANCA)-associated vasculitis [37]. In ANCA-associated vasculitis, CD28 was found to be downregulated on circulating and lesional CD4^+^ T- cells [46] and CD4^+^CD28^-^ T-cells have been described as the major source of pro-inflammatory cytokines (particularly IFN-γ and TNF-α) in Wegener’s Granulomatosis [47]. These authors have also shown that the severity of the disease was positively correlated with the increased proportion of CD4^+^CD28^-^ T-cells. Therefore, the proportion of CD4^+^CD28^-^ T-cells in untreated ENL patients needs to be investigated.

The analysis within ENL group has revealed that the median percentage of CD4^+^ T-cells was significantly decreased during and after prednisolone treatment in each ENL patient. On the other hand, the median percentage of CD8^+^ T-cells was significantly increased following prednisolone treatment unlike CD4^+^ T-cells. Interestingly, the CD4^+^/CD8^+^ T-cell ratio was significantly deceased after prednisolone treatment implying the role of prednisolone in re-establishing the immune homeostasis by downregulating excessive CD4^+^ T-cell activation. Studies have shown that prednisolone reduces CD4^+^ T-cells [14, 48]. A reduction of CD4^+^ to CD8^+^ T cells ratio in patients with rheumatoid arthritis when as little as 2.5mg of prednisolone was administered every 6hrs has been reported [49]. Mshana et al. were the first to hypothesize that ENL is precipitated by an imbalance of T-lymphocyte subpopulations [50]. According to this hypothesis, ENL has two phases: initiation, due to an imbalance in T-cell subpopulations with decreased suppressor cells (now called T_regs_) and perpetuation. Hence, the finding of the imbalance of T-lymphocyte subpopulations (CD4^+^/ CD8^+^ ratio) in this study would support the initiation of ENL reaction in patients with lepromatous leprosy. The initiation of ENL reaction by hyper activation of T-cells further explained by the association of viable bacterial load with the release of soluble antigenic material at the site of bacterial degranulation in ENL patients compared to the intact bacteria observed in non-reactional LL patients [51, 52]. Hence, it seems that unlike in the LL patients, macrophages in ENL patients may be activated and processes the bacteria and hence fragmented and granular bacterial deposits seen in these patients. The activation of macrophages by T-cells (most likely through IFN-γ secretion) may produce inflammatory cytokines such as TNF-α and IL-1β [53] which could amplify the immune hyperactivation and hence tissue damage in ENL patients.

### Increased IL-17 producing T-cells in untreated ENL patients

We confirmed that untreated patients with ENL had a significantly higher median percentage of IL-17 producing T-cells than LL patient controls. T-cell subset analysis has shown that patients with ENL had a higher percentage of IL-17 producing CD4^+^ T-cells than LL patient controls before treatment. On the other hand, the percentage of IL-17 producing CD8^+^ T-cells was not significantly different in both patient groups. After prednisolone treatment, none of these cells show significant difference.

Th17 cells have been identified as a new subset of the T- helper cells and as potential mediators of inflammation associated with various autoimmune and mycobacterial diseases [27]. Th-17 produces IL-17 and it has been reported that IL-17 plays a key role for activation and recruitment of neutrophils to the site of infection in inflammatory diseases [54]. IL-17 is considered as a pro-inflammatory cytokine because it increases IL-6, IL-8, nitric oxide, TNF-α and IL- 1β production by various cell types. Th17 cells are the least studied T-cells in leprosy and only few studies have indicated the involvement of Th-17 in the immunopathogenesis of ENL [20, 26, 28]. The involvement of IL-17 as pro-inflammatory cytokine in human inflammatory diseases such as rheumatoid arthritis, psoriasis, crohn’s diseases, systemic lupus erythematosus, inflammatory bowel diseases and multiple sclerosis has been reviewed by Miossec [55]. It has been described that Th17 and T_regs_ have reciprocal functions. We identified increased IL-17 producing T-cells in untreated ENL patients compared to LL patient controls. Analysis of IL-17 producing T-cell subsets has shown that IL-17 producing CD4 T-cells are significantly increased in active untreated ENL patients and diminished after prednisolone treatment which signifies the importance of CD4 T-cells in the pathogenesis of ENL. Hence, understanding the exact role of IL-17 in ENL reaction will benefit the development of novel immune modulators that reduce inflammation and thereby protect tissue damage in patients with ENL.

### Untreated ENL patients had similar proportion of CD25 but reduced FoxP3 expression compared to LL controls

This study has shown that the level of CD25 expression on CD4^+^ T-cells was not significantly different in patients with ENL and LL controls before treatment but the expression of CD8^+^CD25^+^ was lower in patients with ENL than in LL patient controls. This could be explained by the fact that CD25 is not only expressed on T_regs_ but also on activated T-cells as previously described [56]. Hence, it is not appropriate to compare the level of phenotypic expression of CD25 only on either CD4^+^ or CD8^+^ T-cells in patients with ENL and LL controls since the expression of CD25 could have different implications in these groups: activation in patients with ENL reaction and regulation in non-reactional LL patients.

Although transient FoxP3 expression in ex-vivo activated human T-cells has been reported [57], it remains as a good marker for T_regs_. In this study, for immunophenotyping of T-cell subtypes, unstimulated PBMCs were used and hence our FoxP3 result is less likely to be over represented or activated. The stimulation of CD4^+^CD25^–^ human T-cells have shown to generate CD4^+^CD25^+^ T cells which can also express FoxP3 [58]. The present result shows that the percentage of CD4^+^FoxP3^+^ T-cells in the PBMCs from patients with LL controls was more than twice the percentage of CD4^+^FoxP3^+^ T-cells in the PBMCs from patients with ENL before treatment. However, a similar percentage of CD4^+^FoxP3^+^was obtained in both groups after treatment. On the other hand, the expression of FoxP3 in CD8^+^ T-cells was not significantly different in both groups before and after treatment. To obtain a more characterized FoxP3 population that has a regulatory property, CD127 was used in conjunction with CD25 as an additional marker as previously described [59]. Therefore, the T_regs_ described here are better characterized than previous reports and thus, our present finding of T_regs_ is more refined than previous studies.

Similarly, the expression of CD25 by CD4^+^ and CD8^+^ T-cells was not significantly different before and after prednisolone treatment within ENL group. This can be explained by the fact that CD25 can be expressed by activated as well as regulatory T-cells as previously described [58]. Therefore, although the CD25 expression in CD4^+^ or CD8^+^ T-cells before and after treatment is comparable, it may not have the same role i.e. it could play with activation role before treatment and a regulatory role after treatment in the same patient. However, this needs to be verified by functional assay of these cells.

Interestingly, the expression of FoxP3 in CD4^+^ T-cells was significantly increased after prednisolone treatment within ENL group. Thus, unlike the expression of CD25 in CD4^+^ T-cells, prednisolone upregulates the expression of FoxP3 in CD4^+^ T-cells and hence increases tolerance through immune suppression [14, 39]. On the other hand, the expression of FoxP3 in CD8^+^ T-cells was not significantly different before and after prednisolone treatment. Hence, it appears that prednisolone does not affect the expression of FoxP3 in CD8^+^ T-cells in these patients.

### Conclusion

It has shown that T_regs_ may protect from non-specific memory T-cell activation and potential tissue damage [60]. Hence, the reduced frequency of CD4^+^T_regs_ and the increased CD4^+^/CD8^+^ T-cells ratio in untreated patients with ENL may explain the possibility of induction of excessive immune activation owning to the pre-existing high load of bacterial antigens in patients with lepromatous leprosy. We found that a significant reduction of the percentage of CD4^+^ regulatory T-cells and an increased percentage of CD4^+^/CD8^+^ T-cell ratio and IL-17 producing T-cells in untreated patients with ENL compared to the non-reactional LL patient controls. These findings suggest that ENL is associated with a reduced percentage of regulatory T-cells and increased CD4^+^/CD8^+^ T-cell ratio as well as IL-17 producing T-cells. This immune imbalance could lead to the initiation of ENL reactions either by permitting increased production of antibodies critical to immune-complex formation or as a cell-mediated immune response in patients with lepromatous leprosy.

## Supporting information

S1 Fig Gating strategy for CD4^+^ Tregs in unstimulated PBMC. Acquired events were first gated using a forward scatter area (FSC-A) versus height (FSC-H) plot to obtain singlets. Subsequently, the events were subjected to a lymphocyte gate through Side scatter area (SSCA) versus Forward scatter area (FSCA). After gating for CD3^+^ T-cells, a Boolean gate platform was used to obtain CD3^+^CD4^+^CD25^+^FoxP3^+^CD127^-/0^ cells.
(PPTX)

S2 Fig Gating strategy for CD8^+^ Tregs in unstimulated PBMC. Acquired events were first gated using a forward scatter area (FSC-A) versus height (FSC-H) plot to obtain singlets. Subsequently, the events were subjected to a lymphocyte gate through Side scatter area (SSCA) versus Forward scatter area (FSCA). After gating for CD3^+^ T-cells, a Boolean gate platform was used to obtain CD3^+^CD8^+^CD25^+^FoxP3^+^CD127^-/0^ cells.
(PPTX)

S3 Fig Gating strategy for CD25 and FoxP3 expression on CD4^+^ and CD8^+^ T-cells in unstimulated PBMC. (PPTX)
