# Supplementary figures and images for: T-cell regulation in Erythema Nodosum Leprosum
Source: PLoS Negl Trop Dis. 2017 Oct 9;11(10):e0006001. doi: 10.1371/journal.pntd.0006001 (PMC5648259; doi:10.1371/journal.pntd.0006001)

## Slide 1
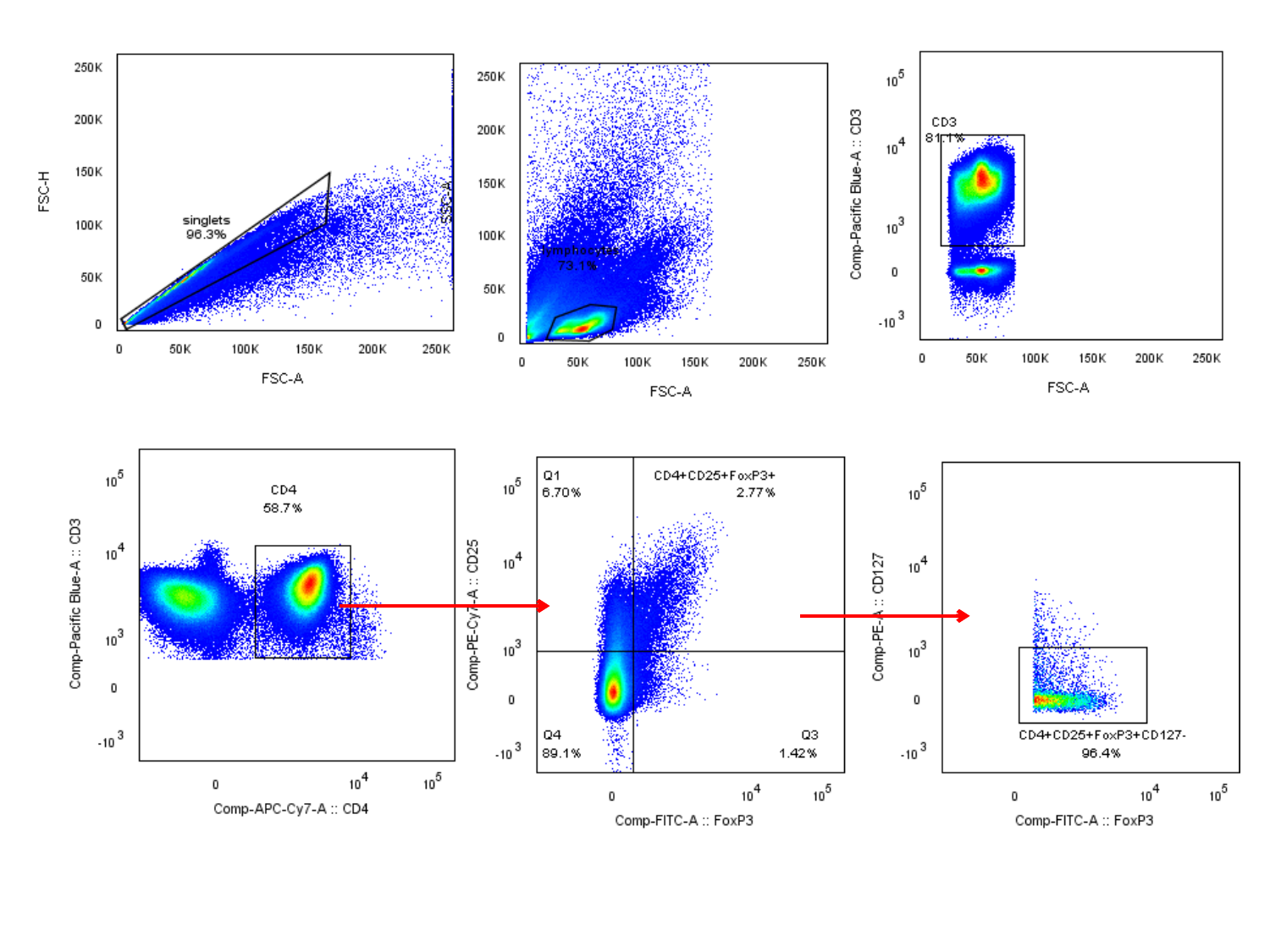

Supplement: S1 Fig — Acquired events were first gated using a forward scatter area (FSC-A) versus height (FSC-H) plot to obtain singlets. Subsequently, the events were subjected to a lymphocyte gate through Side scatter area (SSCA) versus Forward scatter area (FSCA). After gating for CD3+ T-cells, a Boolean gate platform was used to obtain CD3+CD4+CD25+FoxP3+CD127-/0 cells. (PPTX) [file pntd.0006001.s001.pptx]

## Slide 1
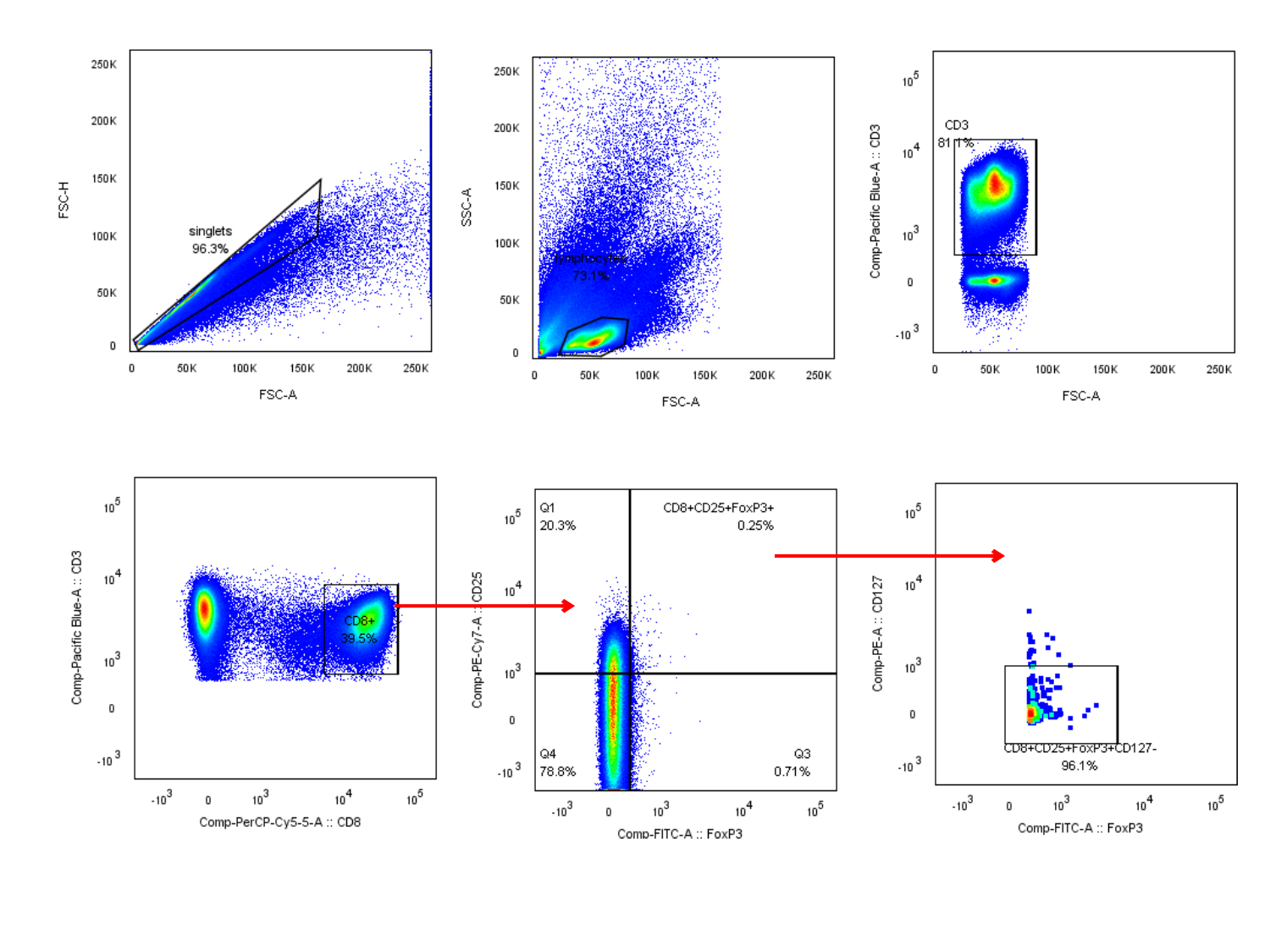

Supplement: S2 Fig — Acquired events were first gated using a forward scatter area (FSC-A) versus height (FSC-H) plot to obtain singlets. Subsequently, the events were subjected to a lymphocyte gate through Side scatter area (SSCA) versus Forward scatter area (FSCA). After gating for CD3+ T-cells, a Boolean gate platform was used to obtain CD3+CD8+CD25+FoxP3+CD127-/0 cells. (PPTX) [file pntd.0006001.s002.pptx]

## Slide 1
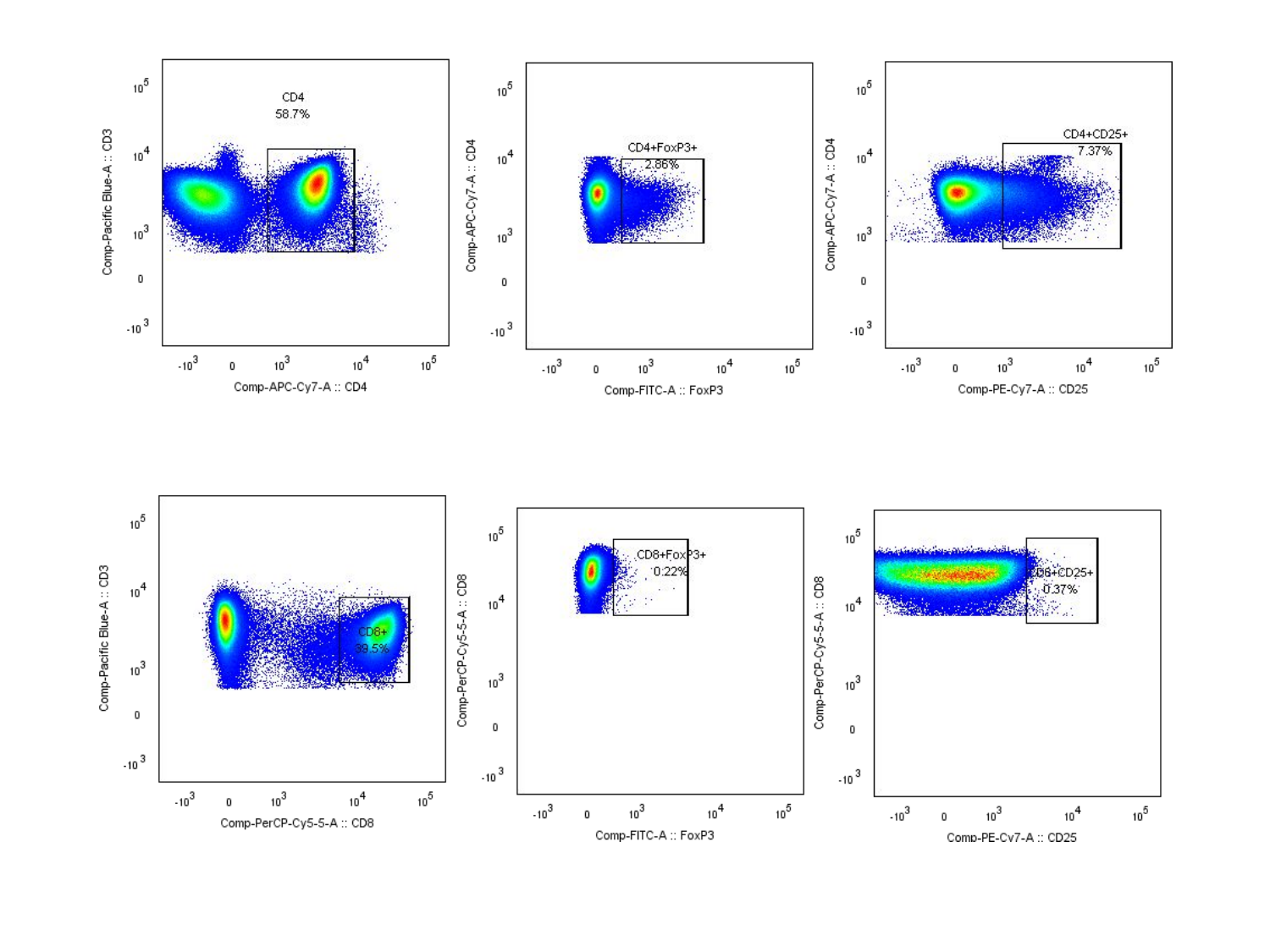

Supplement: S3 Fig — (PPTX) [file pntd.0006001.s003.pptx]
